# Masochist or Murderer? A Discourse Analytic Study Exploring Social Constructions of Sexually Violent Male Perpetrators, Female Victims-Survivors and the Rough Sex Defense on Twitter

**DOI:** 10.3389/fpsyg.2022.867991

**Published:** 2022-06-23

**Authors:** Chelsea-Jade Sowersby, Marianne Erskine-Shaw, Dominic Willmott

**Affiliations:** ^1^Department of Psychology, Faculty of Health and Education, Manchester Metropolitan University, Manchester, United Kingdom; ^2^Division of Criminology, Sociology and Social Policy, School of Social Science and Humanities, Loughborough University, Loughborough, United Kingdom

**Keywords:** rough sex, rough sex defense, sexual violence, victim-blaming, Twitter, social construction

## Abstract

“Rough sex” can be considered an act of sexual violence that is consensual or non-consensual, often resulting in bodily harm and in rare cases, fatalities. The *rough sex defense* is typically advanced by male perpetrators in an effort to portray a sexual encounter as consensual, to avoid criminal sanctions for causing injury or death. Public attitudes toward this defense are often reflected on social media following high profile cases and appear to echo dominant discourses that reinforce widely held sexual violence stereotypes. Therefore, this study aims to deconstruct public attitudes surrounding the rough sex defense. Namely, how female victims/survivors and male perpetrators of sexual violence are constructed online, whilst exploring the wider implications upon society. NVivo12 NCapture software was used to collect a sample of 1000 tweets mentioning the terms “rough sex” or “rough sex defense.” Data were examined using Feminist Critical Discourse Analysis (FCDA), underpinned by a social constructionist perspective, to elicit emergent discourses. Findings indicate that Twitter allowed women to resist harmful victim-blaming discourses and constrained binary identities. Opposingly, men were constructed as sexually entitled predators, yet resisted these subject positions by advocating support for male victims/survivors. Additional analyses examine account holders’ constructions of British Parliamentarians (MP’s) and their campaigns against the rough sex defense. These constructions demonstrated a cultural, heteronormative and victim-blaming understanding of sexual violence, which calls for legislative clarity.

## Introduction

International crime statistics display the increasing prevalence of sexual violence globally. Since 2014, countries including Canada, Australia, New Zealand, and Ireland have all experienced year on year increases in police recorded sexual crime (United Nations, 2018), while US statistics reveal the same upward trend for rape offenses, with annual increases observed since 2013 (Federal Bureau of Investigation, 2018). In the year ending March 2019, more than 58,000 rape allegations were reported to police in England and Wales – the highest figure since records began (Office for National Statistics [ONS], 2019). At the time of writing, the most recent data for England and Wales show a slight reduction in reported rape offenses of 0.7% for the year ending March 2020 (Office for National Statistics [ONS], 2021). These figures are disconcerting though rising rates of reported sexual victimization may be driven, in part, by enhanced awareness of historical abuse cases via mainstream and social media (DiBennardo, 2018), as well as the emergence of widespread online campaigns such as the #MeToo movement; developed to raise awareness and support for victims of sexual violence (Bogen et al., 2019; Hindes and Fileborn, 2019). Indeed, following the rise of the #MeToo movement, globally reports of sexual victimization have increased (Alaggia and Wang, 2020; Willmott et al., 2021). In England and Wales alone, the onset of specialist police operations that investigated allegations of historic sexual abuse perpetrated by high profile entertainers such as Jimmy Savile, have led to a sharp increase in police recorded sexual offenses since October 2012 (Burnett and Smith, 2017). Alongside changes in the way police and the Home Office record sexual offenses, part of the increase in sexual victimization can be explained by what has become known as the “Savile effect.” For instance, following the high-profile exposure and condemnation of offenses committed by individuals like Jimmy Savile, and apparent police willingness to investigate such historic allegations, both male and female survivors of sexual abuse appear more willing to report their victimization to authorities (Office for National Statistics [ONS], 2018, 2021). As such, the communication around and perceptions toward sexual violence, as portrayed on social media platforms, may be beneficial in aiding understanding of current, influential discourse around the topic.

Whilst acknowledging that both men and women experience sexual violence, reported crime figures do consistently highlight the gendered nature of such crimes. Globally, men are overwhelmingly the perpetrators of sexual offenses and women and girls most often those victimized (World Health Organization [WHO], 2013: Willmott et al., 2021). In England and Wales alone, recent reported crime figures reveal that 98% of those prosecuted for the most serious sexual offenses were male, with females accounting for 84% of those victimized (Crown Prosecution Service [CPS], 2019). However, as it is universally agreed that only a small proportion of sexual offenses are ever formally reported to the police, true prevalence rates are extremely difficult to ascertain and often underestimated. It is also important to recognize that prevalence figures are influenced by vast underreporting of sexual offenses experienced by men, due in part to the stigma that still surrounds male sexual victimization and perceived social expectations surrounding masculine norms (Mathews et al., 2015; Depraetere et al., 2020; Douglass et al., 2020; Gough and Peace, 2020; Weare, 2021). Indeed male-on-male rape was not formally recognized by legislation in England and Wales until 1994, with this and recent research highlighting the range of alternative social misconceptions and pressures that serve to discourage men who are sexually victimized from reporting their experiences (Hine et al., 2021; Weare, 2021; Murphy et al., 2022). Clearly, it is important to consider both the gendered nature of the law and male survivor voices which highlight barriers to reporting their abuse when interpreting the accuracy of prevalence rates and subsequent prosecutions. Nonetheless, given that women are also known to substantially under report their sexual victimization, available reported crime statistics and national crime survey data that take into account sexual offences that have not been formally reported to the police, continue to indicate the pervasiveness of sexual violence perpetrated by men, toward female victims (Maas et al., 2018; D’Avanzato et al., 2021).

Alongside concerns surrounding the prevalence of abuse against women, recent media coverage of numerous high profile sexual crimes that resulted in the victim’s death, have thrust the issue of women’s safety into public consciousness. Media coverage which often precedes vast public discussion on social media. Considerable theorizing and research now exists that attempts to explain such sexual violence and aggression (Johnson and Beech, 2017; Blagden et al., 2018; Willmott et al., 2018; Chan, 2021; Vera-Gray et al., 2021), with notable Forensic Psychologists such as the late Dr. Ruth Mann, devoting entire careers in pursuit of effective interventions that reduce and prevent sexual recidivism (see Mann and Rollnick, 1996; Mann, 2004, 2011, 2016; Mann et al., 2019). The consequences of sexual offending on victim-survivors and families are also understood well (Boduszek et al., 2019; Chowdhury et al., 2021; Debowska et al., 2021; Duncan et al., 2022; Sharratt et al., 2022). A plethora of studies have aided understanding surrounding public attitudes toward sexual violence generally (Bows and Westmarland, 2017; Debowska et al., 2018; Hudspith et al., 2021; Smith et al., 2021), though to date, very little research has explored public attitudes toward more specific types of sexual violence, resulting in harm and fatality as a consequence of “rough sex.”

### “Rough Sex”

Generally, research distinguishes between rough sex as consensual sexual behaviors and sexual violence as non-consensual sexual behaviors (Burch and Salmon, 2019). Indeed, the term “rough sex” is traditionally understood to refer to a range of sexual activities that whilst involving a degree of force or aggression, are nonetheless consensual (Eastman-Mueller et al., 2021). Yet as Gallagher et al. (2022) point out, more recently the term rough sex has taken on a dual definition, frequently conceptualized as referring to non-consensual violence and aggression used during either consensual or non-consensual sex. Research has also assimilated sexual violence with behaviors such as: bondage, discipline, dominance, submission, sadism, and masochism (BDSM) which are widely used consensual sexual practices. BDSM is a behavioral construct encompassing a consensual subculture of expressed intent, violent fetishism, and bodily harm whereby those with sadomasochistic tendencies are satisfied through acts such as restraining, choking, slapping, verbal humiliation, whipping, and subjugation. Sadism can be defined as sexual gratification derived from the infliction of physical pain on another individual (i.e., power). Opposingly, masochism involves sexual gratification experienced while being subjected to physical pain or humiliation by another individual (i.e., powerlessness) (Lines, 2015). Both concepts, allow individuals to express sexual agency, however, such terms are arguably socially constructed (Stabile et al., 2019), and their perceived meaning is likely to alter between contexts and individuals (Burch and Salmon, 2019). Such meanings are therefore important in our understanding of gendered power relations and unequal sexual spaces including within intimate relationships, politics, pornography and the criminal justice system (Smith and Skinner, 2017; Stabile et al., 2019; Eaton and McGlynn, 2020). For example, one issue within research and public discourse is that definitions of rough sex and sexual violence each incorporate overlapping sexual behaviors, with unclear distinctions between the two. Here, distinctions made are often based around whether “rough” sexual behavior is consensual. Likewise, the boundary between rape and consensual sex is precarious and permeable for some in certain situations, as are the boundaries between rough sex and abuse/trauma. These definitions blur the lines between safe non-normative sex and an offense against the person being committed. Recent investigation of ways in which mainstream pornography contribute to such blurred perceptions and indeed misconceptions surrounding what constitutes consensual sexual and sexual violence, found clear evidence that sexually violent acts are routinely presented as normative sexual behaviors. Analysis of more than 130,000 titles accompanying individual pornographic content from three popular websites, displayed in excess of 15,000 videos (12%) depicted acts of sexual violence (Vera-Gray et al., 2021).

It is important to note that not all BDSM practices involve violence and should not be characterized as deviant or problematic behaviors. Rather, many enjoy engaging in such behaviors in a mutual, safe, consensual manner (Burch and Salmon, 2019). However, in relatively rare cases, engagement in rough sex (consensual or non-consensual) have led to fatalities. For example, 43 guilty verdicts of homicide of a woman or girl during rough sex, were identified between 2000 and 2018 (Yardley, 2021). Although instances of fatality during rough sex are rare, some evidence indicates that cases are rising (a tenfold increase between 1996 and 2016; We Can’t Consent To This, 2020). In such cases, there is a growing inclination for defendants (accused of causing physical injury or death during violent sexual encounters) arguing that their actions were the result of accidental injury or death sustained during consensual rough sex. However, blurred boundaries and definitions make it difficult to differentiate and prove beyond reasonable doubt at trial that guilt has been established and thus a criminal offense has been committed. Doubts are also evident in public discourse, with speculation of consensual rough sex narratives perpetuated to reduce culpability (Edwards, 2020). Such discussions infer changing social constructions of rough sex, across contexts, and within law.

### The Rough Sex Defense

Edwards (2016) argues that the rough sex defense is a criminal defense plea advanced by a criminally responsible perpetrator following the murder of their sexual partner during or immediately after rough sex has occurred. In an effort to diminish criminal liability and receive a reduced sentence or punishment, the perpetrator alleges the intent was in aid of sexual gratification through consensual sexual violence (Edwards, 2016). A defense is presented, which often involves the construction of a consenting hypersexual victim-survivor with atypical sexual preferences and a promiscuous sexual history (Bows and Herring, 2020). In a recent widely publicized case, British backpacker Grace Millane was killed on December 2nd 2018, during a sexual encounter with a man she met online, whilst traveling around New Zealand. Despite ultimately being convicted of her murder, the defendant, Jesse Kempson, presented a “rough sex gone wrong” defense at court where he claimed consent had been obtained. This case has generated vast commentary and opinion in mainstream and social media, presenting an opportunity to better understand public attitudes toward this type of sexual violence more broadly.

In some instances, the rough sex defense is utilized by defense lawyers to position women as culpable for their own murder (provocation) due to the assumption that consent mitigates accountability (Edwards, 2016); a narrative that may persuade juries to determine not-guilty verdicts. It is important to note however, that not all individuals who stand trial are indeed guilty. In addition, defense teams present the rough sex defense in an effort to reduce sentence length for those found guilty. The invalidation of sexual violence can lead to secondary victimization of victims/survivors during their interaction with the criminal justice system that often involves exposure to insensitive language and their victimization experiences constructed as false, unreliable, or vengeful (Smith and Skinner, 2017; Stabile et al., 2019). Whilst trial discourse has been widely studied in the context of rape, few studies have explored discourse used to construct the rough sex defense online, where public perspectives and debates are frequently advanced. Current law in England and Wales states that a defense of consent to defend bodily harm is not sufficient according to the *Domestic Abuse Bill* (Edwards, 2020). This would prevent the alleged consent of the victim/survivor from being used as a defense to prosecution. However, an apparent loophole has been identified and exploited. Some defendants are using consent as a defense for sexual violence during casual sex on a first date, which therefore deviates from the definition of “domestic abuse” as set out in the Bill. On 6th July 2020, British parliamentary members (MPs) proposed a statutory amendment to the *Domestic Abuse Bill* (2020). The amendment sought to prevent those accused of causing bodily harm during sexual activity including serious injury or a victim’s death, from relying on a victim’s consent to the infliction of this harm, as a viable defense (Edwards, 2020). The law applies in all circumstances in England and Wales and is not limited to those which might also encompass incidents of domestic abuse, meaning that consensual “rough sex” is no longer a possible defense for though who have caused an individual’s death during a sexual encounter. This amendment was granted royal assent on 29th April 2021. Exploring these discussions in the public domain, allows for more nuanced understanding of how sexual violence and rough sex are socially constructed online, while also navigating the complexities of perceived consent, and use of such consensual arrangements to mitigate serious harm.

### Consent and Intent: British Legislation

Currently, the only legislative definition of consent in England and Wales is contained within the sexual offense statute (Sexual Offences Act, 2003). Here consent can only be established where a complainant had both the *freedom* and *capacity* to make a choice about whether to partake in a given sexual act at a specific point in time. The need for consent is somewhat compounded by the requirement that a defendant need only have a *reasonable belief* in consent. Something which is often difficult to ascertain (Hindes and Fileborn, 2019) and increasingly so based upon the complexity of the offense where sexual and physically violence acts intersect. Consent is often underpinned by a combination of verbal communication and non-verbal communicative behaviors. In fact, Wignall et al. (2020) found British university students often assumed or negotiated consent based largely on interpretation of non-verbal cues. Considered alongside the frequency with which rough sex and sexual violence is presented as a normative sexual behavior on popular pornography platforms (Vera-Gray et al., 2021), beliefs about consent to “rough sex” may well be obtained through interpretation of non-verbal information and based largely upon pre-existing sexual scripts that are frequently ill informed and problematic. Sexual scripts are individual criteria for appropriate sexual behavior, which provides a socially constructed framework for how to process our interactions during sexual conduct. The interpretation of such scripts are integral to how we understand such closely connected offenses (against the person and sexual) that often overlap and share similar behaviors (bodily harm during rough sex and rape). Here, it is important to note that not all rough sex is non-consensual, harmful or traumatic for women and therefore, consideration of pre-existing sexual scripts and better understanding how consent is and can be obtained, is key to supporting both the victim and the accused.

Women utilize their gendered sexual scripts of what they deem acceptable and unacceptable and often choose to consent to such acts, taking full acceptance of their sexual partners intentions (bodily harm) to receive sexual gratification. Alternatively, sexual scripts may result in assumed consent, where it has not been verbally implied thus leading to the potential for non-consensual sex. Therefore, consent is ambiguous and the individual differences surrounding the interpretation of these sexual scripts can affect the treatment of victims/survivors in court, as well as the accused. For example, society often categorizes sexually violent encounters as having an ideal victim/survivor versus a monstrous offender (DiBennardo, 2018), and it is possible that more ambiguous consensual situations, will lead to less credible perceptions of the victim. However, although some description of consent is provided in the Sexual Offences Act (2003), there is arguably no statutory definition of consent for fatal and non-fatal offenses against the person involving rough sex because legislation states that one cannot consent to bodily harm (Dunkley and Brotto, 2019). This creates a loophole of uncertainty around rough sex because laypeople often believe that if consent to inflict bodily harm during sex is obtained, this means rough and violent sex is legal and morally acceptable. However, in such cases, an offense against the person may well have occurred if intent to cause harm is ascertained. As such, although consent is clearly an important feature here, the importance of intent should not be underestimated when assessing whether a criminal act has been committed during rough sex.

Legally, criminal offenses require the existence of both *mens rea* and *actus reus*. Mens rea is the psychological aspect of a crime, having the knowledge that one’s action (intentional) or lack of action (negligent) would cause a crime to be committed. Actus reus denotes the wilful participation or failure to perform an act, which causes harm. This applies whether bodily harm, or death, occurred due to sadomasochistic interests of both parties. Legislation is explicit that if assault occasioning actual bodily harm (Offences Against the Person Act, 1861, s. 47) or grievous bodily harm (Offences Against the Person Act, 1861, s. 20) was intended, foreseeable, and consensual, the actus reus and mens rea requirements are likely satisfied, and thus a basis for prosecution. Therefore, there is no plausible defense to intended assault resulting in injury, unless the activity involved is one which prosecutors or parliament recognize to have positive social benefits, such as tattooing, surgery, and contact sports (Bows and Herring, 2020). This deems it acceptable to agree to (intended) bodily harm during these activities, but not sexually transgressive practices. Although this provides some understanding of how the rough sex defense can be steered via intent to harm, to reduce sentencing or culpability, it is also important to note that not all rough sex activities result in bodily harm and are mostly permissible in court (Weinberg, 2016). Importantly, as British legislation around consent and intent during “rough sex” is currently unclear, and ambiguous, perpetrators and defense lawyers may be more likely to advance narratives which endorse victim blaming, rape mythology and minimize sexual violence, when in pursuit of a lesser criminal charge, making the constructions around this defense plea worthy of further exploration.

### Performative Gender Roles in a Sexually Violent Discursive Climate

The rough sex defense is problematic as it often reproduces gendered hierarchies by drawing upon typical masculine (controlling, demanding, aggressive), and feminine (submissive, obedient, passive) gender roles (Lines, 2015). “Slut-shaming” is the tradition of punishing a sexually liberal woman by tarnishing her reputation and branding her as undesirable (Hackman et al., 2017). This is often a tactic used by defense teams to victim-blame women and involves delivering a defense based on women’s sexual history and preferences, alcohol consumption and clothing. Slut-shaming can have damaging implications, such as influencing the treatment of victims/survivors within society, court, and police interviews (Hackman et al., 2017). This highlights the importance of researching gender-role stereotypes in sexual violence, as it is an unequal sexual space. Discourses are value-laden and often reflect the interests of dominant groups within society, yet burdens those with less power, as powerful social pressures subordinate women (Lazar, 2017; Stabile et al., 2019). Often, constructions of femininity provide sexual violence victims/survivors with a limited range of discourses, as female sexuality is bound with an expectation of passivity (Bogen et al., 2019). It can be argued that the public dialogues and social attitudes around appropriate sexual behavior is a form of benevolent sexism and relies on heteronormativity. Indeed, the law takes a gendered approach, being bound in gender-specific terminology. For example, the legal definition of rape in England and Wales stipulates that woman cannot be perpetrators and instead need protecting from harm during sex. This is despite alternative evidence that displays women can and do display recurring sexually aggressive behaviors that cause physical and psychological harm toward men (Douglass et al., 2020; Weare, 2021). Arguably, suggestions that women are not capable of sexual aggression serve to actively promote the narrative that women can never consent to BDSM and thereby reinforce similar gender stereotypes. Specifically, sexual violence victimization is often constructed using damaging victim-blaming discourses, such as rape-myths. Rape myth culture is based on stereotypical cognitive distortions, misconceptions and assumptions, which endorse or excuse sexual violence against women using concepts such as “men have needs” or “sexual urges that are difficult to control” (Stubbs-Richardson et al., 2018). Hindes and Fileborn’s (2019) analysis examined how myths surrounding sexual consent were accepted and reproduced by news media following the #MeToo movement. Most reports perpetuated stereotypical, and thus, limited understandings of gender roles underpinning sexual violence, with men seen as naturally aggressive pursuers of sex. Clearly there remains a need for research exploring the multi-faceted subject positioning of sexual victimization and perpetration (Burch and Salmon, 2019).

Male perpetration of sexual violence is often constructed as biologically based and reinforced through masculine discourses (Taylor, 2020). More specifically, men are given the powerful subject position of seeking sex and being the primary inflictor of pain (Mathews et al., 2015; Edwards, 2016). Several feminist authors have criticized how dominant, masculine ideologies enable patriarchal behaviors (rape myth acceptance, slut-shaming, victim-blaming defense pleas) which influence public dialogue and consequently, court cases outcomes (Lines, 2015; Hindes and Fileborn, 2019; Rowlands and Walker, 2019; Stabile et al., 2019). Rough sex can be constructed as having healthy, eccentric sexual fetishes (acceptable) or as domestic abuse, rape, and sexual assault (problematic). Clearly, there are numerous contradictory gendered meanings that cohere around rough sex, sexual violence, and the rough sex defense. It is important to conduct research on the shifting and competing discourses that are continually produced and reproduced, as these can contribute to feminist efforts to deconstruct pervasive, gendered power-relations within the online realm (Stubbs-Richardson et al., 2018; Hindes and Fileborn, 2019).

When exploring both perceptions of rough sex, and sexual offenses, it is important to consider the role of sensationalist reporting in media outlets, which are likely to influence gendered discourse around the topic (DiBennardo, 2018). Crimes which violate cultural and social norms are generally more likely to result in sensationalist reporting, which receive more interest from the public and are likely to evoke fear and disgust (Dowler, 2006). Harper and Hogue (2014) found that media accounts of sexual offenses are communicated more emotionally, negatively and aggressively, compared to other crimes. Media (including social media) representation of sexual violence is largely focused on the female victim, yet a “predator” discourse of the offender/accused has become central to how people conceptualize and discuss sexual violence. Undoubtedly this results in the vilification of sexual offenders and social outrage. As such, certain harmful discourses which demonstrate ideologies around female victims and male perpetrators, also have problematic implications for the accused. For example, the condemnation of male sexual offenders contributes to the reinforcement of rape myth acceptance, as male perpetrators of sexual offenses are often represented as monstrous, predatory and aggressive in news media (DiBennardo, 2018). On the other hand, female sexual offenders and victims are negotiated through benevolent sexism perspectives, positioning female perpetrators as over-sexualized, thus pariahs to femininity (Kilty and Bogosavljevic, 2019), and female victims as passive, weak and helpless (Schwark, 2017). Alternatively, public discussion via social media (e.g., Twitter and Facebook) portray victims as having agency (often called survivors) which tends to evoke public admiration and appeal to broader audiences. However, much media research focuses on victim framing only, without examining how it may shape representations of offenders and the implications of this (DiBennardo, 2018). To better understand potential gendered power relations and public discourse around rough sex and the rough sex defense, it is necessary to consider the representations of both victim and accused.

### Digital Feminism: The Resistance of Sexual Violence on Twitter

Harmful sexual violence media depictions are prevalent within films, music, pornography, literature, and society (Burch and Salmon, 2019). The media distorts and romanticizes sexual violence against women as a means to navigate plot-lines and shape public perceptions. This is problematic as it can hide male-initiated domestic abuse and sexual assault (Edwards, 2016). Public discourse around rough sex and sexual violence heavily influence how sexual violence is discursively framed and gender biased in legislative decisions and reform, however, public opinions can be solely based on misunderstandings, and thus relying on lay people’s opinions to reform legislation is potentially problematic (Smith et al., 2021). Moreover, Stubbs-Richardson et al. (2018) explored how rape culture, victim-blaming and slut-shaming was constructed through sexual assault cases in mainstream media; Twitter. They argued victim-blaming discourses were frequently used by males to position women as responsible for their own rapes. Currently, through the use of social media platforms, such as Twitter, a single victim-blaming tweet can negatively influence attitudes that contribute to widely held harmful constructions of consent, intimacy, and pleasure. Notably, that women are less worthy of pleasure (Stubbs-Richardson et al., 2018). Subsequently, many feminist authors established the importance of exploring online perceptions to deconstruct harmful discourses and share marginalized perspectives (Maas et al., 2018; McCauley et al., 2018). Cultural representations of gender roles and sexual violence are also present within the criminal justice system, as decision makers within these systems contribute to the same problematic beliefs, by operating a system which fails to prioritize the needs of victims/survivors. There is therefore a clear need for more internet-mediated research to consider the influence that public perceptions have within policy and practice (Maas et al., 2018).

Currently, the rough sex defense has grown in importance in light of recent activist Twitter accounts “@wecantconsentto” and “@countingdeadwomen” campaigning against such declarations being used in court. The rough sex defense is a term devised by campaigners and the media to promote activism. Whilst this coverage may appear informative, Twitter reproduces misconceptions, such as cases of rough sex are frequently justified and that perpetrators often do not receive a conviction which undermines the severity of such acts. This reproduction of misinformation becomes clouded as current law around bodily harm is not easily understood or accessible to laypeople (Bows and Herring, 2020). This may have a detrimental influence on whether victims come forward or not.

Several internet-mediated research studies have documented how social media platforms such as Twitter offer an opportunity to assess real-world applications of victim-blaming that might otherwise be difficult to obtain (Maas et al., 2018; Stubbs-Richardson et al., 2018; Bogen et al., 2019; Stabile et al., 2019). Therefore, sexual violence victims/survivors may access Twitter to disclose problematic experiences when they feel distrustful of support, are not yet ready to disclose in-person, or have support accessibility issues (Bogen et al., 2019). It is noteworthy that much work in this area focuses on analyzing semi-structured interviews or mainstream media reporting, often neglecting widely used platforms such as Twitter (Mathews et al., 2015; Burch and Salmon, 2019; Hindes and Fileborn, 2019). Such work is important in understanding how sexual violence is represented and discussed in media, with consideration given to the perception of consent, and existing gendered power relations. For example, Hindes and Fileborn (2019) utilized a post-structural feminist framework to explore the representation of sexual violence and consent in news media reporting of a high-profile case (in relation to the #MeToo movement). The Aziz Ansari (the accused) case concerned the pursual of aggressive sexual advances from the Aziz Ansari, the male comedian and actor, in spite of verbal communication opposing sex, from the female victim. Findings suggested a limited understanding of sexual violence, and normalizing of coercion, with little consideration of varying presentations of consent (or non-consent). Furthermore, harmful gendered stereotypes were reproduced in the news reporting, portraying men as naturally aggressive and ascribing responsibility to women to protect themselves against sexual advances. The findings provide important insight into how sexual violence is represented, and how such representations may be navigated through the use gender stereotypes and power relations. However, such news reports are conducted by journalists, and involve sensationalist reporting aimed to evoke strong emotional responses. It is possible that discourse around sexual violence in open, public spaces (online) will differ as discussions are uncensored, can be anonymous, and are constructed by lay people, rather than journalists. In this regard, less is known about how Twitter may be used to perpetuate sexually violent attitudes, which in turn normalize and fail to address problematic sexual behaviors underpinning sexual violence perpetration. Therefore, this internet-mediated research is worthy of further analysis (Stubbs-Richardson et al., 2018). The limited research that have explored discourses around sexual violence on Twitter have enhanced the understanding of how victims and perpetrators of sexual violence are represented (Stubbs-Richardson et al., 2018), and the reaction to disclosures of sexual violence (Bogen et al., 2019) from public posts and discussions online. However, previous work has largely utilized content analysis which, while informative, limits the in-depth understanding of discourse, and the interpreted purpose, and gendered nature of such discourse. Additionally, less is known of how individuals navigate the complexities of consent around rough sex, which may lead to harm and fatality. In an attempt to facilitate the understanding of online discourse surrounding sexual violence victimization and perpetration, the following aims were generated: to explore public attitudes of sexual violence and the rough sex defense and to consider what implications the accepted and resisted subject positioning have upon public attitudes, policy and practice. Also, to explore how male rough sex perpetrators, and female rough sex victims/survivors are discursively positioned on Twitter.

## Materials and Methods

### Data Collection

Research denotes social media platforms are a social construction of reality. Social media such as Twitter facilitates interactions and the transmission of emerging issues, whilst providing researchers with a valuable resource to help analyze social constructs such as sexual violence (Sloan and Quan-Haase, 2017). Twitter was selected as it is unrestricted, contains naturally occurring data, and is geographically and topically diverse (Maas et al., 2018; McCauley et al., 2018). Tweets represent social, cultural and historical meaning-making processes which shape individual attitudes and beliefs toward social phenomena. The media then influences these attitudes, as individuals share such discourses online. The search terms and social constructs “rough sex” and “rough sex defense” were chosen as opposed to hashtags. Using search terms eliminated the possibility of confirmatory bias, as hashtag use often reproduces a homogeneous sample of tweets and thus, biased content depending on how it is constructed (Maas et al., 2018). Data was collected in a non-invasive way, from readily accessible, public accounts, whereby account holders’ data can only be collected if users have their profiles set to public (British Psychological Society, 2017; D’Avanzato et al., 2021). Firstly, an anonymous academic Twitter account was created prior to data collection, which allowed the maintaining of researcher safety. QSR International Pty Ltd’s (2020) NCapture extension for the NVivo12 qualitative software was selected to collate data. NCapture is a Google Chrome extension that allows researchers to collect tweets directly from Twitter. The rough sex search-term elicited 13,859 results, and the rough sex defense search-term elicited 10,659 tweets over a 7-day data collection period from 01/07/2020 to 07/07/2020, 1.5 years after the sensationalist media reporting of Grace Millane’s murder and just as parliament were campaigning for the statutory amendment to legislation. Gathered tweets were filtered to ensure they met the inclusion requirements and did not include exclusionary factors. The first 500 tweets per search-term (1000 in total) were harvested for interpretation, which was consistent with previous literature (Maas et al., 2018; McCauley et al., 2018; Stubbs-Richardson et al., 2018; Bogen et al., 2019). Next, tweets were maximally anonymized before coding by replacing usernames with “account holder 1.” Contextual information was provided to interpret the tweets, whilst still anonymizing identifiable, sensitive, and harmful information. For example, names of MPs and specific job positions were replaced with “[male/female MP].” This data collection method was appropriate as it may increase the likelihood of uncovering honest accounts and attitudes around sexual violence victimization and perpetration, as individuals are likely to communicate opinions due to perceived anonymity and security of online spaces (Bogen et al., 2019).

### Inclusion and Exclusion Criteria

A strict inclusion and exclusion criteria were necessary to ensure a unique but representative data set. The inclusion criteria involved collecting any unit of meaning, provided the tweets were written in English, and contained the search term “rough sex” or “rough sex defense.” However, the exclusion criteria involved: eliminating retweets (non-original discourses), non-English tweets, spam tweets (consisting of corrupted links), tweets that were uninterpretable, tweets with identical content from the same account holder, and finally, tweets which may have had potentially damaging effects for account holders, as this avoids unfair practice of breaching safeguarding policies (British Psychological Society, 2017).

### Epistemological Position

According to social constructionism, it is assumed that knowledge and reality is socially produced through networks of discourse (Burr, 2015). Narratives are performative (masculinity and femininity) and consequently, discourses invite some behaviors (male-initiated sexual violence) and discourage others (female sexual liberation) (van Dijk, 2015). This analysis unpacks discourses which position males and females in particular ways in relation to rough sex and the broader realm of heterosexuality. The focus is on how power, and performative gender roles are reproduced, negotiated, and contested within online talk. Hence the need for analysis, which illustrates privileged and oppressed perspectives (Sloan and Quan-Haase, 2017; Wigginton and Lafrance, 2019). The present internet-mediated research explored how wider implications such as gender asymmetries, structural injustices, and perpetrator-versus-victim/survivor identities were legitimized, renegotiated, and resisted within language to socially construct male perpetrators, female victims/survivors of rough sex, sexual violence itself and the rough sex defense on twitter.

### Analytic Strategy

Data analysis was completed using feminist critical discourse analysis (FCDA), underpinned by social constructionism to explore the discursive positioning of sexual violence, male perpetrators and female victims and the rough sex defense (Burr, 2015; van Dijk, 2015; Lazar, 2017; Wigginton and Lafrance, 2019). By thorough reading of the sample, the lead author became intimately familiar with the data before classifying tweets into themes. 52 initial themes were systematically chunked whereby each theme represented something noteworthy, such as a descriptive code. The 52 themes were organized into theme files, and through reanalysis it was recognized some themes overlapped. Therefore, some themes were collapsed or merged with another. For example, the “women should man up,” “women lack communication,” and “women are overreacting” themes were merged into “victim-blaming.” A final sample of 28 *in vivo* themes were identified. Then, three superordinate and two subordinate themes were selected for closer analysis. Relationships between or within these discourses were then established, evidencing outliers using pink arrows or connections using green arrows. For instance, within the subordinate theme of “men get assaulted too,” different themes were recorded, such as “women use consent for entrapment” and “deceptive women report false allegations,” which were tensioned with “he needs mental health counseling.” This continued until marginalized perspectives and disparities between intricate subject positions were accounted for. Ethical approval was granted by the Psychology Department Research Ethics Committee at the host institution Manchester Metropolitan University.

## Results and Discussion

As demonstrated in Table 1, three superordinate themes and two additional subordinate themes were deployed between account holders, based on their perceptions of female sexual violence victims/survivors, male sexual violence perpetrators, and the rough sex defense. The first superordinate theme, ‘Reproducing the Boring “feminazi” versus “pain slut” Dichotomy’ suggested that women were categorized based on their sexual preference and such categorizations were constructed with negative, defamatory language such as “slut” versus “boring.” A further subtheme of “Rough Sex Fixes Female Flaws: Women Accepting the Problematic Subject Position” describes how women also reproduced discourses around feeling ostracized by society’s beauty standards and often reproduced harmful labels of feeling “disordered,” “unattractive” and in need of fixing. Rough sex was constructed, by women, as a tool to renegotiate societies ideologies around female sexuality and menopause. ‘Entitled “men and their excuses” Reproducing the “bullshit victim-blaming” Narrative’ was the second superordinate theme identified through analysis. Here, a juxtaposed discursive labor was presented, whereby Twitter posts framed manhood as prescribing sexual entitlement, and notions pertaining to victim-blaming were evident in such discourse. A subordinate theme, “‘Men get assaulted too”: a Transgression of Hegemonic Masculinity’ further found male account holders often positioning themselves as victims to withstand the notion that only women can be traumatized during sex, challenging male perpetrator stereotypes. Importantly, whilst considering the wider implications of online interactions this study concluded that female MPs were vilified for campaigning for change, whereas male MPs were constructed as dedicated and were applauded for their work. These unequal power relations and patriarchal constructions made up the third superordinate theme of “policy and practice require reform” (see Table 1).

**TABLE 1:** Coded superordinate and subordinate themes.

Themes	Coding	Selected illustrative extract
**Superordinate:** Reproducing the boring “feminazi” versus “pain slut” dichotomy	Women were constructed as one out of two undesirable subject positions: working-class “pain slut[s]” or middle-class boring “feminazi[s].”	91. My boomer dad saying ‘you’re really turning into a…is it a feminazi?’ in response to me explaining to my mum what the ‘rough sex’ bill is, is just a whole bag of irony innit.
**Subordinate:** Rough sex fixes female flaws: women accepting the problematic subject position	Men deployed blended discourses of irrationality to position women as broken, which invalidated women’s trauma. Women were in acceptance of this subject position.	423: Realized today in some texts w my therapist that my “daddy kink” and appreciation for rough sex were trauma based and that’s why I don’t like any of it anymore because I’m actually processing the trauma wow wild
**Superordinate:** Entitled “men and their excuses” reproducing the “bullshit victim-blaming” narrative	Women were rejecting problematic subject positions by constructing men as sexually entitled and using victim-blaming to mitigate culpability.	11: Rough sex without your consent, when you say it hurts or ask him to stop and he continues or even worse is rape sis. Please leave because he will get worse and you should be scared. You’re not safe.
**Subordinate:** “Men get assaulted too”: a transgression of hegemonic masculinity	Men transgressed from typical masculinity and renegotiated perpetrator stereotypes by positioning themselves as vulnerable to victimization.	147: See alot of guys like that, but alot of females take consent back and THAT is what we are afraid of. Being labeled a rapist after having rough sex. Some of yall use it as a trap too. Its dangerous for a man. I was accused at 13. Fucked me up, im 20 and still can’t get over it.
**Superordinate:** Policy and practice require reform	Female MPs were ostracized. Meanwhile, male MPs were applauded. Policy and practice problematically reproduced binary and heteronormative understandings of gender and sexuality.	989: [female MP] There is no “rough sex defense” how foolish of you. You’ve been a disgrace to the [political] Party and working people for years. Resign please.

### Reproducing the Boring “Feminazi” Versus “Pain Slut” Dichotomy

*‘Geez I definitely wouldn’t call it grotesque. I’ve watched him for a long time and don’t remember him saying that. Just wondering about rough sex not full bdsm. I guess pain slut was the wrong word to use. My bad.’* (Account holder 139)

The first superordinate theme pertained to derogatory discourses:

*“Bitch”* (Account holder 381),

*“Pain slut”* (Account holder 139),

*“pussy”* (Account holder 842),

“*Ass made for rough sex”* (Account holder 369)

Such terminology is clearly deployed to objectify and disadvantage women. This is acknowledged by account holder 139’s apology of *“my bad.”* This female account holder is expressing sexual agency by sharing her fantasy of seeing a male create specific pornographic content. The account holder had previously labeled a particular woman as a *“pain slut”* before apologizing. *“Slut”* discourses position women as undesirable and categorizes women based on their sexual activity. This demonstrates women were being subjected to positions of vulnerability by implying they should be treated like animals and sexual objects. This was also found in Gough and Peace’s (2000) study whereby men denied women their human status, by constructing them as animals in order to assert dominance.

Despite Stubbs-Richardson et al. (2018) sample illustrating a predominantly victim-supportive response to sexual victimization, victim-blaming discourses were notable by their majority within this analysis. Several account holders problematically constructed women who prefer soft sex as: *“vanilla,” “feminazi,” “weak bitch,” “neoprude,”* and needing to *“man up.”* This problematizes women’s preferences and portrays women as boring and feeble. The current findings are consistent with Bogen et al.’s (2019) analysis which found victim-blaming discourses, informed by rape myths and stereotypes, were reproduced online when discussing sexual violence. This demonstrates female sexual violence victims/survivors are vilified in comparison to male sexual violence perpetrators.

*‘There’s a new tik tok trend that’s absolutely disgusting to me. it is teen girls showing there bruises after having “extremely rough sex” it’s not my place to talk about other people’s sex lives but honey your arm being broken isn’t just rough sex. that’s abuse.*’ (Account holder 452)

Additionally, in the above extract the female account holder appears to be gatekeeping what is and what is not acceptable for females to consent to by positioning young women as vulnerable. This account holder attempts to police women’s bodies through positioning young girls wearing bruises as proudly displaying them like a trophy on social media which in turn, perpetuates non-normative sex as unacceptable, with little consideration of consensual acts. The account holder conflates bruises and broken bones in order to emphasize their point, possibly with the intention to gatekeep. Whilst such gatekeeping may signify concern and disapproval of behaviors resulting in harm, and protection of peers engaging in similar behaviors, it is important to consider that motivations behind such discourse are likely to differ across groups. For example, findings from Vaillancourt and Sharma (2011) suggest that women are intolerant and feel negatively toward other females who signify sexual availability and promiscuity. For this reason, females are more likely to use indirect aggression such as ostracism and negative criticism to devalue and desexualize other women who they perceive to be potential rivals (intrasexual competition). In short, this evidence suggests that women are threatened by, disapprove of and punish women who act promiscuous. Aside from gatekeeping explanations, the account holder constructs rough sex as abusive and dangerous which may suggest that gender ideologies are perpetuated online through online trends. Here, patriarchal power is resisted by highlighting that broken bones are not “on-trend” or acceptable during sex. This evidence shows that the media mainstreams contradictory meanings, which cohere around attitudes regarding intimacy.

Within this analysis, rough sex was suggested to be something men *do* to women in order to inflict harm and prove their dominance. This was connected to medical discourses female account holders deployed around their sexual injuries:

*“broke a nail,” “torn,” “tender labia,” “cervix is bruised up,” “earrings being ripped out,” “splits you open,” “hurt and sore,” “nose bleed,” “hip problems,” “unable to walk,” “bit a chunk out of her tit,” “vaginal bleeding,” “slashed with knives,”* and *“strangled.”*

These discourses framed women as victims of traumatic assaults and long-lasting injuries. These account holders frame rough sex in such a way to highlight the disproportionate level of injuries in which women sustain as a result of rough sex. This is contrasted with Burch and Salmon’s (2019) results, whereby women demonstrated their sexual agency by framing rough sex as pleasurable and constructed sex as fun and worthwhile of minor injuries. Account holder 341 resisted the sex-as-punishment narrative by positioning themselves derogatively as a *“slut”* and deserving of rough sex, whilst framing rough sex as desirable. This clearly demonstrates not all account holders construct rough sex as sexual violence in all cases. This demonstrated that feminine expectations of passivity were replaced with sexually liberal repertoires. This was also found in Hindes and Fileborn’s (2019) study who found emergent discourses around women’s sexual liberation were prominent. Conversely, women were using Twitter to construct sex as primarily for male enjoyment, with their desires placed at the forefront of the encounter, this extract also conforms to the male culturally validated hypersexuality narrative. Whereas women are constructed as sexual/domestic puppets, whom are to be sexually available upon request. This construction serves the purpose of sharing the idea that women should adhere to sexual scripts of subordination.

*‘Be a WIFE is full time to be a cook, nurse, and therapist, on top of being your husband own personal whore when he want you to be, a stripper when he wanted to spice shit up. A bitch that has no problem with rough sex’* (Account holder 357)

Furthermore, women were dichotomized by various account holders; middle-class, female MPs (who discussed rough sex ending in harm as problematic), were problematized as being a *“nanny state,” “prude bint,”* and an *“angry bitter woman with a dry vagina.”* Female MPs were depicted as menopausal, therefore sexually undesirable for intruding on people’s private lives and policing sexual desires and experimentation. Rather, few account holders reproduced the subject position of working-class, *“council estate girls,”* which were bound with many damaging, classist, and stereotypical ideologies, such as: “*drinking Stella Artois,”* having *“tats and [a] tan” “[wearing] fake designer brands,”* and using *“swear words and slang”* within their speech. This was similar to Stubbs-Richardson et al.’s (2018) findings of the virgin-whore binary, which women were restricted to. In turn, this suggests women are offered one of two constrained identities, as they cannot experience the subject position of being an upper-class, *“prude bint”* as well as a lower-class, tattooed, *“pain slut,”* both of which are problematic and undesirable. Interpretive repertoires reflected androcentric definitions of womanhood, whilst constructing women as being ideologically feminine, nurturing, and domesticated. Such discourse is indicative of benevolent sexism; characterizing and positioning women using positive language, which simultaneously signifies weakness and inferiority to men. Account holder 357 echoes society’s expectations of how a married woman should behave. Similar patriarchal prescriptions of ownership were notable, present more overt sexism;

*“Her husband’s personal whore”* (Account holder 357)

Here women are positioned as sexual objects, whilst blended discourses of *“stripper”* reinforced sexist assumptions that women should be sexually available without challenge. Several studies highlighted sexually violent male attitudes reinforced female disadvantage, whereby men blamed sexual aggression on women’s flaws as a cook, mother, and housekeeper (Stubbs-Richardson et al., 2018; Debowska et al., 2019). This suggests problematic dominant ideologies are based on male privilege and the objectification and domination of women. Therefore, this analysis supports that damaging gender-role attitudes are prevalent online.

*“Ok so I’m justifying rape by claiming I never seen it and all hardcore scenes don’t perpetuate rape it’s just rough sex lol. And the company doesn’t promote it I never seen a pornhub ad of someone getting raped ur starting to reach for straws that’s not there chill out”* (Account holder 158)

Account holder 158 was putting forward the argument that hardcore pornography does not perpetuate rape and highlights the differences between pornography and rape, as the two are often conflated online. This was connected to sexual violence being reduced to *“just rough sex”* by account holder 158. The salient discourse *“just”* minimized and justified sexual violence encounters as hardcore, yet consensual. *“Chill out”* and “*reach for straws that’s not there”* demonstrated denial and handed women the melodramatic subject position. Hindes and Fileborn (2019) elicited similar findings whereby tendencies to minimize sexual violence also connected to a broader culture that excused sexual violence against women. Indeed, given that Vera-Gray et al.’s (2021) extensive recent research displayed the frequency with which non-consensual sexual violence were presented as a normative consensual sexual behavior on three of the most popular pornography platforms (including the website Pornhub denoted by account holder 158 above), such discourse and positioning is indicative of the pre-existing distorted sexual scripts that are exposure to such content contributes toward (Eaton and McGlynn, 2020). Comparably, many account holders provided potentially problematic discourses around victims/survivors by suggesting they should *“learn self-defense,”* and *“know ur worth”* which positioned sexual violence victims as responsible for the violence they have experienced. It may be assumed that such advice is well-intended, and it is possible that narratives of this kind are driven by fear induced from media depictions of national sexual violence events which are often sensationalized, evoking negative and anxious emotions (Dowler, 2006; Harper and Hogue, 2014; DiBennardo, 2018). However, such discourses feed into victim-blaming narratives and present victims/survivors with an ideological dilemma, suggesting sexual violence is problematic, however, it is the victim’s behavior which warrants change, as opposed to perpetrator behavior. Assumptions that women do not have the tools to prevent sexual violence, such as confidence, self-defense, and strength individualizes the blame and further perpetuates sexual violence. This finding was similar to Hackman et al. (2017) who found being a victim/survivor was viewed as the result of not taking initiative to protect oneself from danger. Alternatively, examples of digital feminism such as *“love yourself first,”* and *“#useyourvoice,”* are examples of victim-supportive discourses, offering victims ideas of how to protect themselves amidst safety concerns. Consequently, women are confronted with a double-standard of being expected to take control, protect themselves and be strong which classic, male-defined femininity does not typically permit.

#### Rough Sex Fixes Female Flaws: Women Accepting the Problematic Subject Position

A subtheme further demonstrated a paternalistic and reductionist narrative, constructing women as damaged, whilst implying women’s disabilities, mental-health issues, and past trauma were easily fixable by men through the means of rough sex. Likewise, female account holders were in acceptance of this misogynistic, and difficult-to-resist rhetoric.

*“There was one guy who said he liked rough sex while choking the girl, I said I wasn’t comfortable with that all and when he asked why, I told him it triggered my PTSD. He told me I was crazy and stupid for living my life to the maximum and that I was overreacting.”* (Account holder 343)

Account holder 343 recounts a conversation she had with a male when setting sexual boundaries. Their focus is based on the male’s reaction when she had explained sexual choking was a mental health trigger and his response was to reproduce discursive stigma around mental health by making her feel inadequate and labeling her *“crazy.”* Diagnostic discourses and harmful feminine generalizations, such as “crazy,” discursively blended sexual violence victims and women as being biologically flawed:

*“Bipolar”* (Account holder 200)

*“Depression”* (Account holder 44)

*“PTSD”* (Account holder 343)

These appeared to be oppressive tactics males use to ascribe power to themselves, whilst framing women as problematic and psychologically unstable. This reductionist viewpoint of victimization also strips legitimacy away from women’s trauma by medicalizing their feelings. In addition, this depicts women’s trauma as an overreaction, which conforms to restrictive and emotionally unstable, female gender stereotypes. Interestingly, whilst discourse surrounding opposition to requested rough sex suggests produced depictions of a flawed or *“crazy”* female, research by Dunkley and Brotto’s (2019) found that men held the assumption that female involvement in BDSM reflected symptoms of psychopathology (opposed to their opposition to rough sex). As such, women involved in consensual rough sex leading to (unintended or non-consented) harm may be represented negatively. Together, both present and previous work (Dunkley and Brotto, 2019) suggest online damaging victim-blaming discourses may be prevalent across situations, and such discourses need deconstructing.

Society reinforces that bodily changes in women which occur due to disability, menopause and age are undesirable. Some women choose to reject this rhetoric by sharing their valid desire to feel attractive again through sexual liberation, aiming to destigmatize female sexuality and menopause. Alternatively, numerous female account holders accepted the subject position of feeling disordered by framing themselves as in need of sexual escapism to deal with difficulties, such as: *“chronic disease,” “young menopause,” “stress”* and menstruation problems. Here, rough sex was framed as a coping mechanism for women with a range of medical, psychological and cultural strains. This illustrates women were mostly combating the stigma of being labeled “disordered” by society’s standards. Until systemic problems are deconstructed within research, society will continue to allow perpetrators to individualize the blame to female victims, forcing them to shoulder the burden of victimization instead of penalizing sexual violence perpetrators. Society questions sexual violence victims more so than offenders and categorizes victims’ actions as inadequate. Furthermore, discourse around consensual rough sex often appeared to be negotiated and explained through female’s self-disclosure of mental and physical health difficulties, thus “excusing” the “need” for rough sex, and self-positioning as someone to be fixed, or requiring escape.

### Entitled “Men and Their Excuses” Reproducing the “Bullshit Victim-Blaming” Narrative

The second superordinate theme identified male entitlement as an accepted subject position, prevalent across a large selection of tweets. The following narratives predominantly navigated sexual violence rationalizations.

*“Bec[ause] of circumcision, when I hit my late 30s I noticed a dramatic decline in sensitivity due to the inevitable keratinization from the glans being exposed my whole life. I had to start having very rough sex in order to feel pleasure and achieve climax”* (Account holder 366)

Account holder 366 highlights valid feelings of men aging and the effects of medical procedures such as circumcision which can affect sexual performance and pleasure. In particular constructing men as biologically sexually aggressive, sexually driven, and entitled to women’s bodies:

*“I had to start having very rough sex in order to feel pleasure and achieve climax”* (Account holder 366)

This mitigates criminality by suggesting it is within men’s biological nature to demonstrate aggression and hypersexuality. Hindes and Fileborn (2019) elicited similar aggression-supportive attitudes, which reinforced the male sexual drive discourse, positioning men as predators, unable to control themselves, with women being the prey. This could be argued to be a clear employment of male power aimed to deflect responsibility onto human biology. This finding was similar to Gough and Peace’s (2020) findings whereby repertoires of biology were commonly invoked by men as a way of authorizing gender difference as “naturally” correct. The positioning of men as naturally aggressive may further perpetuate rape myth narratives (Depraetere et al., 2020) and are likely to influence both the perception and reporting of male victims. Moreover, it is important to consider that twitter user 366 is presenting valid feelings around their inability to achieve climax which could be argued as an intention to engage public discussion around stigmatized life events such as circumcision and delayed ejaculation. This serves the purpose of destigmatizing bodily occurrences when men age. In this case, the narratives surrounding the “need” for rough sex is similar to that of female account holders, who attempted to explain their engagement in rough sex as a response to medical difficulties.

Many account holders portrayed entitlement as being at the forefront of their sexual encounters, *“Everything is about YOU and YOUR needs”* and *“Was he ever offered rough sex?”* evidenced that males were positioned as sexually entitled. The discourse *“had to”* highlighted male supremacy by positioning male pleasure as primary and female autonomy as secondary. These discourses also framed women as responsible for pleasuring a man, and consequently, depicted women as flawed if a man’s sexual desires were not fulfilled. Alternatively, few account holders drew upon harmful assumptions, such as “*bitch likes to be beat and tied up”* and *“[women enjoy] being throat fucked,”* which framed men as subscribing to their male duty to subjugate. This was replicated in Hackman et al.’s (2017) study whereby sex was viewed as a male expectation, and was bound with assumptions, which ought to be challenged.

*“You’re so entitled to your gratification that you believe consent cannot be withdrawn halfway through sex. you don’t listen when women say no, stop, or show clear signs of discomfort because everything has to be about YOU and YOUR needs.”* (Account holder 845)

The above extract is a female account holder expressing discontent with a male account holder, tending to generalize the discussion around intimacy to all males. This depicted men as entitled to harm due to female expectations of passivity. This means subordinate feminine ideologies are problematic in that women believe they restrict women’s bodily autonomy and liberation. Many account holders constructed men as forceful, *“I asked him numerous times to stop”* and *“you don’t listen when women say no”* constructed female safety as secondary to male sexual gratification. This represents the power imbalance between male perpetrators and female victims/survivors. This was similar to studies which found myths, assumptions, stereotypes, and biased discourses were reproduced within male defense pleas, sexual assault trials, and rape jury conclusions to position women as responsible for sexual violence (Stubbs-Richardson et al., 2018). Therefore, in practice we must deconstruct defamatory discourses, which perpetuate sexual violence and manipulate trial outcomes.

A large selection of account holders reproduced rationalizations of sexual violence perpetration. More specifically, the slut-shaming rhetoric that promoted sexual aggression and suggested sexually liberal behavior in women causes sexual violence victimization.

*“Rough sex with many men”* (Account holder 403)

*“She wanted it”* (Account holder 393)

It is also possible that such comments from female account holders suggest gatekeeping in the form of slut-shaming by other women to protect their peers from engaging in certain behaviors (Vaillancourt and Sharma, 2011).

Account holder 862 framed BDSM discourses as an *“alien”* concept which mitigated male responsibility and placed the blame on unfamiliar and inaccessible BDSM language. Here, men were assigned the subject position of being uneducated, which demonstrated male assumptions regarding consent can be harmful. Supporting evidence suggested sexually active women frequently received negative, slut-shamed reputations, meanwhile men were excused for sexual assertiveness and such behavior was reinforced by rape myths (Hackman et al., 2017). This illustrates how gender stereotypes perpetuate an oppressive double-standard.

“Well there are various reasons, but first we’ll come back to the kink/situational part of things. R*pe fantasies and those of the like are not uncommon in the slightest and aren’t a bad thing. They’re typically related to kinks such as ‘surprise’ and rough sex, not getting + “ (Account holder 131)

Account holder 123 constructed himself as nurturing and sensitive, *“kiss all the bruises”* and *“massaging where she’s sore,”* which manipulated and masked the aggressive intent of bodily harm to achieve sexual pleasure. This is a discursive tool men employ to alternatively frame sexual violence as negligent, as opposed to an intentional act. Other account holders suggested *“sex can go wrong,”* and *“you weren’t properly raped,”* which reduced sexual violence to vigorous sexual activity and invalidates women’s trauma. Account holder 131 suggests that fetishes, kinks and rape fantasies are common amongst the kink community, therefore are not unacceptable. The above quote deems rape fantasies as acceptable, as fantasies do not indicate that the person will act on such fantasies or pose a risk to society. This account holder positions those who are non-conforming to typical sex as deviant in respect of the law and is rejecting that the law should gatekeep what is and is not acceptable sexual behavior. These framings can contribute to the normalization of rape culture, whilst positioning sexually violent desires as common, therefore acceptable. To reduce rape fantasies to *“not a bad thing”* could be an example of a cognitive distortion which contribute to the perpetuation of rape myth acceptance (Johnson and Beech, 2017; Debowska et al., 2021). Many account holders deployed minimization strategies and denial discourses, such as: “vanilla shit with a sprinkle of r*ape lmao,” “stop creating drama,” and *“no femicide in the United Kingdom.”* These misogynistic attitudes framed women as irrational and reduced sexual violence and bodily harm to an insignificant issue. Similar victim-blaming strategies, informed by rape myths, such as holding harmful assumptions, minimization techniques, and denial were also prevalent within the literature (Hackman et al., 2017; Taylor, 2020). This highlights the misogyny inherent within society regarding men perpetuating, policing, and denying such gendered crime. The discourses here shape understandings of male sexual violation against women as a normal part of men’s behavior and quest for sex, and if these discourses are continually being reproduced by society, victims will never feel listened to. The implications of this may mean that female victims feel devalued and are less trusting in the legislation that serves the purpose of protecting them.

#### “Men Get Assaulted Too”: A Transgression of Hegemonic Masculinity

A further subtheme drew upon discourses which aimed to legitimize advocacy for male victims by rejecting the notion that the law protects all victims. As it stands the law is gendered and heteronormative, which fails to consider the prevalence of male victims. A discursive labor was illustrated, whereby men framed themselves as victims of sexual entrapment, false allegations, and a misandrist and emasculating legislative amendment, which is a real concern for men. However, it could be argued that men are displaying their vulnerabilities in order to highlight the lack of support for male victims and to also re-invent traditional masculine ideologies.

*“Me: Volunteers on a support line for victims of sexual assault, helping both male and female victims and families cope with the aftermath Me: Celebrates the ‘Rough Sex’ defense being made illegal after its use in female murder cases Men: ‘Sexist, Men get assaulted too”’* (Account holder 701)

Throughout this analysis many tweets pertained to positioning women as problematic, for example a “*vindictive ex [who] wants to attack her former partner?”*. Similarly, several male account holders reproduced the male victimization narrative by suggesting they were *“scared,” “afraid,”* and were fearful of *“being labeled a rapist”* for practicing rough sex. In doing so, men were constructed as victims/survivors of false allegations and wrongful convictions. Here, discourses were blended to renegotiate the perpetrator typology, whilst positioning women as irrational, deceptive and manipulative in their sexual violence disclosures. Hindes and Fileborn (2019, p. 12) found similar discursive framings, which positioned victims as “scorned women who weaponize accusations as a means of serving revenge.” The present results are significant because harmful victim-blaming constructions and instilling fear into victims may prevent individuals from identifying and reporting sexual violence including male-on-male victims. Likewise, numerous account holders framed women as the oppressor, *“men get assaulted too”* and *“this woman destroyed me”* are vilifications of perpetrator women, suggesting they receive no punishment as the law excludes male victims. DiBennardo (2018) found that masculinity adds an additional layer to constructions of victim legitimacy. To the extent that as adult men victims fail to live up to masculine, heterosexual ideals, their victim status is devalued similarly to that of adult women. Here, men contest the female victim/survivor typology and appear to deploy minimization tactics by denying alternative or competing versions for example female victim’s trauma. Male account holders were displaying their vulnerabilities through the re-invention of patriarchal scripts, which Gough and Peace’s (2020) findings suggested is a powerful way of stalling change of the present *status quo* to devalue masculinity.

Both account holders were renegotiating typical feminine constructions of victimization/survivorship through defying traditional domestic scripts. This illustrates women were conforming to subject positions and behaviors traditionally denied (dominance). This outcome was contrary to that of Hindes and Fileborn (2019), who deduced most heterosexual disclosures constructed male sexual violence perpetration as acceptable. However, this failed to acknowledge female-initiated sexual violence as problematic. The insights gained from this study may challenge gendered assumptions of sexual violence perpetration in that sexual violence victims can be both male and female. The narrative function of such constructions serves to position men as vulnerable victims, which in some instances, may aim to silence women’s narratives by using the tactic of ‘whataboutism’ the technique of responding to an accusation or stereotype by making a counter accusation or deflecting onto an entirely different issue (Taylor, 2020).

### Structural Injustice Implications: Policy Requires Reform

Whilst exploring the aim of the study to look at the wider implications of public attitudes toward the rough sex defense, this research deduced that these beliefs continued within policy, which made up the third superordinate theme. Many account holders constructed female MPs efforts to campaign as *“a shocking deception,” “flawed,”* and *“projecting juvenile thinking.*” These highlighted discourses were grounded in the assumption that politics are a male domain, unsuitable for women. Again, several account holders suggested *“the state has no place in the bedroom*,” *“[female MPs] just removed even more bodily autonomy from women,”* and *“they may accidentally criminalize kinky sex.”* This constructed the government as draconian and oppressive for policing people’s sexual preferences. This also framed the amendment as an infringement on ethical autonomy, feminism and liberalism. Stabile et al. (2019) found similar constructions within their analysis, whereby policymakers were constructed as providing beneficial policy to already advantaged, positively constructed populations (men), and disadvantageous policy for negatively constructed groups (women) (Stabile et al., 2019). Equally, a variety of account holders positioned female MPs who first introduced the bill such as Home Secretary, Priti Patel as unintelligent through derogatory, damaging and discriminatory labels, such as: *“foolish,” “Thiki patel,”* and *“idiot like you,”* whilst also being instructed to *“resign.”* On the contrary, male MPs were praised and glorified for their work (account holder 799) *“totally determined”* and *“tough territory for a man,”* which demonstrates the female disadvantage embedded within leadership roles. This positioned female MPs as incompetent to work in a predominantly male government. The negative attitudes and criticism toward women, within male-dominated organizations, was also present within Stabile et al.’s (2019) analysis. Contesting these misogynistic narratives means resisting the way in which women are constructed both within and outside policy (Edwards, 2020).

The possible implications for conducting this study at the exact time that MP’s were campaigning for a statutory amendment were that the media was using moral panic to elicit a wider readership and mass hysteria by creating folk devils (individuals, namely men, who transgress from conventional sex). Whilst this may have elicited a bigger twitter sample, the media was responsible for coining the term “rough sex defense” alluding that men were “getting away with murder.” In light of the media recycling old articles regarding Grace Millane, sensationalism tactics would have likely increased fear and elicited strong emotionally weighted responses globally (Dowler, 2006; Harper and Hogue, 2014; DiBennardo, 2018). Further, key information was often omitted such as men being acquitted of committing a sexual offense (although still found guilty of committing an offense against the person). The media selected individual cases of rough sex “gone wrong” and problematically, generalized this to purposefully position women as victims and men as perpetrators. In doing so, cases of male-on-male victims or female-on-male victims were under-represented.

## Conclusion

Feminist Critical Discourse Analysis requires interpretation, but language can be figurative and context dependent (Burr, 2015). Therefore, key discourses could have been missed or misinterpreted due to limitations of knowledge. It could be argued that this study reproduced gendered, individualist, and heteronormative talk by focusing on the dichotomous nature of heterosexual men and women. This highlights the continued need to deconstruct binary understandings of gender and sexuality within policy, by promoting alternative discourses (Hindes and Fileborn, 2019). Therefore, future research should explore the impact binary and heteronormative scripts have on jury decision-making, police interviewing, and LGBTQIA + sexual violence victims/survivors. Likewise, future research should explore how policy discourse contributes to prevailing assumptions about sexual violence victims/survivors. Knowledge of this may challenge gender norms and negative attitudes toward victims both within and outside legislation.

Ultimately, this study addressed the research gap by analyzing social constructions of victims/survivors, and perpetrators, in light of the rough sex defense. Perceptions of sexual violence victimization corresponded to compliance with classist hierarchies, victim-blaming and appropriated male-initiated sexual violence. However, women rejected oppressive, middle-class femininities by deploying sexually liberal repertoires. Alternatively, male account holders used the tactic of ‘whataboutism’ to deviate from traditional scripts by alternatively positioning themselves as victims/survivors to alert society about male victims being further silenced by legislation.

## Data Availability Statement

The raw data supporting the conclusions of this article will be made available by the authors, without undue reservation.

## Ethics Statement

The studies involving human participants were reviewed and approved by Manchester Metropolitan University – HE Faculty Ethics Committee. Written informed consent for participation was not required for this study in accordance with the national legislation and the institutional requirements.

## Author Contributions

C-JS designed the study, did data collection, analyzed the data, and wrote the manuscript. ME-S designed the study and proofread the manuscript. DW wrote and proofread the manuscript. All authors contributed to the article and approved the submitted version.

## Conflict of Interest

The authors declare that the research was conducted in the absence of any commercial or financial relationships that could be construed as a potential conflict of interest.

## Publisher’s Note

All claims expressed in this article are solely those of the authors and do not necessarily represent those of their affiliated organizations, or those of the publisher, the editors and the reviewers. Any product that may be evaluated in this article, or claim that may be made by its manufacturer, is not guaranteed or endorsed by the publisher.
